# CMOS‐Integrated Synaptic Photoreceptor Chip Inspired by Insect Visual Processing

**DOI:** 10.1002/advs.75388

**Published:** 2026-04-21

**Authors:** Jian Chai, Xinyi Xu, Yue Wang, Xiaochen Wang, Hailiang Wang, Yunfei Xie, Xinwei Zhang, Haoyu Wang, Hao Ning, Jiangming Lin, Yongliang Xie, Qihai Jiang, Baoshi Qiao, Xiaolei Ding, Luyao Ma, Shukai Duan, Zhenyi Ni, Huan Hu, Xin He, Fei Xue, Feichi Zhou, Lingfei Li, Srikrishna Chanakya Bodepudi, Rui Yuan, Bin Yu, Yang Xu

**Affiliations:** ^1^ College of Integrated Circuits State Key Laboratory of Silicon and Advanced Semiconductor Materials ZJU‐HIC, Center of CMOS IC Manufacturing Process and Design Zhejiang University Hangzhou China; ^2^ State Key Laboratory of Silicon and Advanced Semiconductor Materials & School of Materials Science and Engineering Zhejiang University Hangzhou Zhejiang China; ^3^ ZJU‐UIUC Institute International Campus Zhejiang University Haining China; ^4^ College of Artificial Intelligence Southwest University Chongqing China; ^5^ ZJU‐Hangzhou Global Scientific and Technological Innovation Center Zhejiang University Hangzhou China; ^6^ Center for Quantum Matter School of Physics Zhejiang University Hangzhou China; ^7^ School of Microelectronics Southern University of Science and Technology Shenzhen China

**Keywords:** Depth perception, dynamic trajectory tracking, SiQDs/ReS_2_ neuromorphic image sensor chip, static feature recognition

## Abstract

Bionic visual processing hardware serves as the core technology for mimicking the efficient information processing of biological vision systems and forms the foundation for perceiving and recognizing both static and dynamic scenes. However, most current bionic visual hardware remains at the level of individual devices and simple arrays. Full hardware implementation of bionic vision chips, as well as comprehensive demonstrations in practical application scenarios, is still scarce. This work proposes a complete optoelectronic insect‐inspired visual sensor. We realize a Si QDs/ReS_2_ heterogeneous integrated neuromorphic chip fabricated using a 180‐nm CMOS process technology and demonstrate its capabilities on both static and dynamic visual tasks. Based on the wavelength‐dependent synaptic properties of ReS_2_, this neuromorphic photoreceptor chip exhibits high‐fidelity discrimination between purple and red features. Through light pulse sequence modulation, it achieves temporal encoding of dynamic visual information and can precisely identify leaf movement trajectories in eight directions. We further achieved 3D perception capabilities, attaining 99.4% accuracy in classification tasks. This work establishes a novel 1D/2D/3D heterogeneously integrated material platform and a CMOS‐compatible integration strategy for a low‐power, multifunctional brain‐inspired neuromorphic sensor chip, advancing the application of optoelectronic devices in artificial intelligence and machine vision.

## Graphical abstract

CMOS‐integrated Si QDs/ReS_2_ synaptic photoreceptor array mimics the parallel processing and wavelength‐selective strategy of insect vision. By combining intrinsic ultraviolet‐violet sensitivity with synaptic plasticity, the chip enables frontend sensory redundancy reduction without external filters, offering a scalable pathway toward lowpower neuromorphic vision systems.

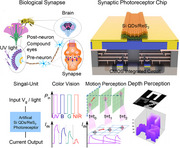

## 1 Introduction

Machine vision is a cornerstone of perception in Internet of Things (IoT) ecosystems, catalyzing advances in autonomous vehicles, smart homes, and industrial robotics [1, 2, 3, 4]. Advanced machine vision systems enable real‐time interaction with external environments, achieving timely and precise responses through accurate data analysis. This capability is particularly crucial in industrial manufacturing processes, especially in high‐speed automated sorting and quality inspection operations [5, 6, 7]. Most contemporary machine‐vision systems adopt a von Neumann paradigm, in which image sensing, memory, and computation are physically separated. This sequential processing paradigm leads to redundant information sampling and data transmission delays [8, 9, 10]. It lacks the capability for front‐end preprocessing and filtering of specific visual features, such as key colors or motion trajectories [11, 12, 13, 14]. This approach leaves complex feature extraction tasks entirely to the back‐end processor, further exacerbating latency and power consumption. Consequently, it struggles to meet the growing demands for high real‐time performance, low power consumption, and edge intelligence [15, 16, 17, 18]. In contrast, insect visual systems efficiently and accurately recognize and process visual information. Taking the honeybee visual system as an example, its spectral perception spans ultraviolet, blue, and green light bands, with relatively low sensitivity to near‐infrared light. Research indicates that bees can precisely distinguish between different colored flowers by identifying unique halos generated by the nanostructures on petal surfaces when excited by ultraviolet light [19, 20]. Furthermore, bees exhibit phototaxis toward ultraviolet light, enabling them to identify flowers from a distance and significantly enhancing foraging efficiency [21]. This visual system also possesses excellent light intensity adaptation, allowing effective target detection under low‐light conditions such as sunrise and sunset, while dynamically adjusting foraging strategies based on ambient illumination. Notably, bees not only recognize static objects but also exhibit extreme sensitivity to fast‐moving targets [22]. This allows them to avoid obstacles in real‐time during flight while precisely locating nectar sources [23]. This bee‐inspired approach, enhancing key spectral features at the front‐end perception stage, efficiently filtering redundant information, and integrating event‐driven processing, provides crucial bionic insights for developing neuromorphic machine vision systems with real‐time processing capabilities and low‐power characteristics.

In recent years, low‐dimensional materials have garnered significant attention in the development of biomimetic visual systems due to their exceptional optoelectronic properties, including unique band structure tunability, strong light‐matter interactions, and outstanding carrier mobility. These materials not only enable high‐quality heterojunction integration without suspension bonds via van der Waals forces but also exhibit excellent 3D stacking compatibility, providing a crucial material foundation for realizing highly integrated, multifunctional neuromorphic vision chips [24, 25, 26, 27, 28, 29, 30]. Among these, semiconductor quantum dots have emerged as a key material system for developing artificial visual devices due to their high light absorption efficiency, spectral tunability, and ease of integration [31, 32, 33, 34, 35, 36, 37, 38, 39, 40]. For example, basic functions such as human‐like eye visual adaptation, color recognition, and feature extraction have been successfully achieved using CNT/CsPbBr_3_‐QD [31], InP DQs/ITZO [32], and M‐QDs/a‐IGZO [33] van der Waals heterojunctions. However, most current research remains focused on device development for single‐application scenarios, and multifunctional integration is required for operation in complex and dynamic environments. Realizing monolithic co‐integration of multimodal sensing with in‐sensor memory computing in a single optoelectronic platform that can operate in real time under variable conditions remains a formidable challenge. Moreover, efficient heterogeneous integration technology between novel intelligent sensing devices and CMOS circuits is crucial for advancing these brain‐inspired devices toward practical applications. Consequently, developing novel brain‐inspired intelligent sensing chips that combine multi‐scenario adaptability with the ability to fully leverage the efficient signal processing advantages of CMOS circuits has become a key direction and urgent need for advancing artificial vision systems.

In this work, we report a photonic neuromorphic device based on Si QDs/ReS_2_ that mimics insect vision, integrated with CMOS circuitry. Due to the capture and release of carriers by defect states at the Si QDs/ReS_2_ interface, the device exhibits remarkable sustainable photoconductive plasticity, effectively simulating insect visual synaptic behavior. This includes photoconductive plasticity across multiple pathways, such as pulse width, interval, number, and frequency plasticity. The device also exhibits selective absorption across different spectral bands, enabling simulation of insect color vision with reliable differentiation between purple and red features. Through modulation of light pulse sequences, it achieves temporal encoding of dynamic visual information, simulating an insect's precise recognition of leaf movement trajectories across eight directions. More significantly, integrating a 16 × 16 Si QDs/ReS_2_ photonic synapse pixel array with CMOS circuits further achieves insect‐inspired 3D perception capabilities, attaining 99.4% accuracy in classification tasks. This research not only validates the feasibility of integrating neuromorphic intelligent sensing hardware with CMOS technology for multifunctional, multi‐scenario applications but also establishes a novel material platform and integration strategy for low‐power, multifunctional biomimetic visual systems. It advances the application of biomimetic intelligent sensing devices in the field of machine vision.

## 2 Results and Discussion

### 2.1 SiQDs/ReS_2_ Artificial Synaptic Photoreceptors

**FIGURE 1:**
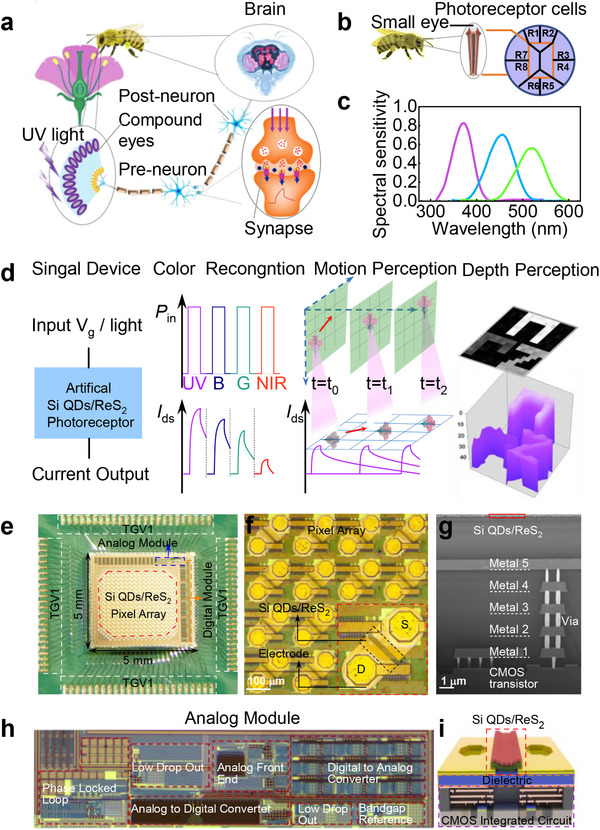
Bionic device concept, structure, and characterization. (a) Schematic of the visual processing pathway and anatomical organization of the honeybee visual system. (b) Cross‐sectional schematic of a single honeybee ommatidium. (c) Spectral sensitivity curves of the Rh4, Rh5, and Rh6 expressing photoreceptors in honeybee ommatidia. (d) Si QDs/ReS_2_ artificial synaptic photoreceptor inspired by the color‐selective and motion vision strategies of honeybees for emulating analogous visual behaviors. Integration of Si QDs/ReS_2_ artificial synaptic photoreceptor arrays with CMOS chips enables on‐chip depth feature recognition. In all panels, *I*_ds_ denotes the photocurrent, UV denotes ultraviolet light, B, G, and R denote blue, green, and near‐infrared light, respectively, and *P*_in_ denotes the incident light intensity. (e) Optical microscope image of SiQDs/ReS_2_ neuromorphic imaging sensor heterogeneous chip. (f) Optical microscope image of SiQDs/ReS_2_ neuromorphic imager chip pixels, with an inset image showing the microscope image of a SiQDs/ReS_2_ pixel; the active region channel width of the pixel is 10 µm. (g) Cross‐sectional scanning electron microscope image of the SiQDs/ReS_2_ neuromorphic imager chip. (h) Microscopic image of the chip analog module. i) Schematic diagram of the 3D structure of heterogeneous pixel and readout circuits in a SiQDs/ReS_2_ heterojunction neuromorphic image sensor chip.

Figure 1a schematically depicts visual information flow in the bee compound eye. Incident light is focused onto photoreceptors (R_1_–R_9_), where phototransduction converts optical stimuli into electrical signals [41, 42]. As shown in Figure 1b,c, the photoreceptor cells of bees exhibit sensitivity peaks in the ultraviolet (∼350 nm), blue (∼440 nm), and green (∼540 nm) bands, enabling efficient extraction of behaviorally relevant cues while attenuating spectral components with lower information content. This spectral selectivity prioritizes detection of UV/blue floral patterns and yields weaker responses to long‑wavelength (red) features, thereby reducing redundancy and improving foraging efficiency [43, 44]. The device exhibits pronounced wavelength dependence, with high sensitivity at 375 nm (UV), 488 nm (blue), and 532 nm (green), and markedly reduced sensitivity at 808 nm (near‑infrared). This spectral profile aligns with the wavelength‑selective strategy of bees, which prioritizes specific bands to efficiently extract behaviorally relevant visual cues. Notably, under the synergistic control of illumination and gate voltage, the photogenerated carriers in SiQDs can be dynamically modulated through the trapping and detrapping processes of interfacial defect states [45]. Based on this mechanism, the device exhibits electrical behavior similar to that of insect visual synapses. Drawing inspiration from the color‐selective and motion vision strategies of honeybees, the device enables efficient extraction of feature and motion information, offering a promising pathway toward front‐end sensory redundancy reduction and low‐power visual processing, as shown in Figure 1d. Furthermore, by integrating a SiQDs/ReS_2_ heterojunction synapse transistor array with a CMOS readout circuit chip, we successfully simulated the multi‐channel composite imaging effect of insect compound eyes. This chip mainly consists of three modules: pixel region, digital, and analog integrated circuits, as shown in Figure 1e. The chip features a resolution of 16 × 16 pixels, with each pixel consisting of a SiQDs/ReS_2_ phototransistor. The active area of each pixel is 10 µm × 100 µm, and the inter‐pixel distance is 50 µm, as shown in Figure 1f. Cross‐sectional scanning electron microscopy images of the Si QDs/ReS_2_ neuromorphic imaging sensor heterogeneous (NISH) chip at the channel position (Figure 1f) confirm the integrated structure of the chip, as shown in Figure 1g. Figure 1h displays Optical microscope images of the chip analog integrated circuit composed of seven sub‐modules: PLL, LDO, ADC, AFE, DAC, and Bandgap Reference (BGR). The functions of each module and the signal transfer process between them are detailed in Note S2. Figure 1i illustrates the schematic cross‐sectional image of the chip. SiQDs/ReS_2_ pixels and chip are vertically interconnected through van der Waals forces, and these interconnections extend directly to the underlying CMOS integrated circuit, facilitating efficient signal transfer and integration.

### 2.2 The SiQDs/ReS_2_ Neuromorphic Imaging Sensor Heterogeneous Chip Enabled by Heterogeneous Integration Technology

**FIGURE 2:**
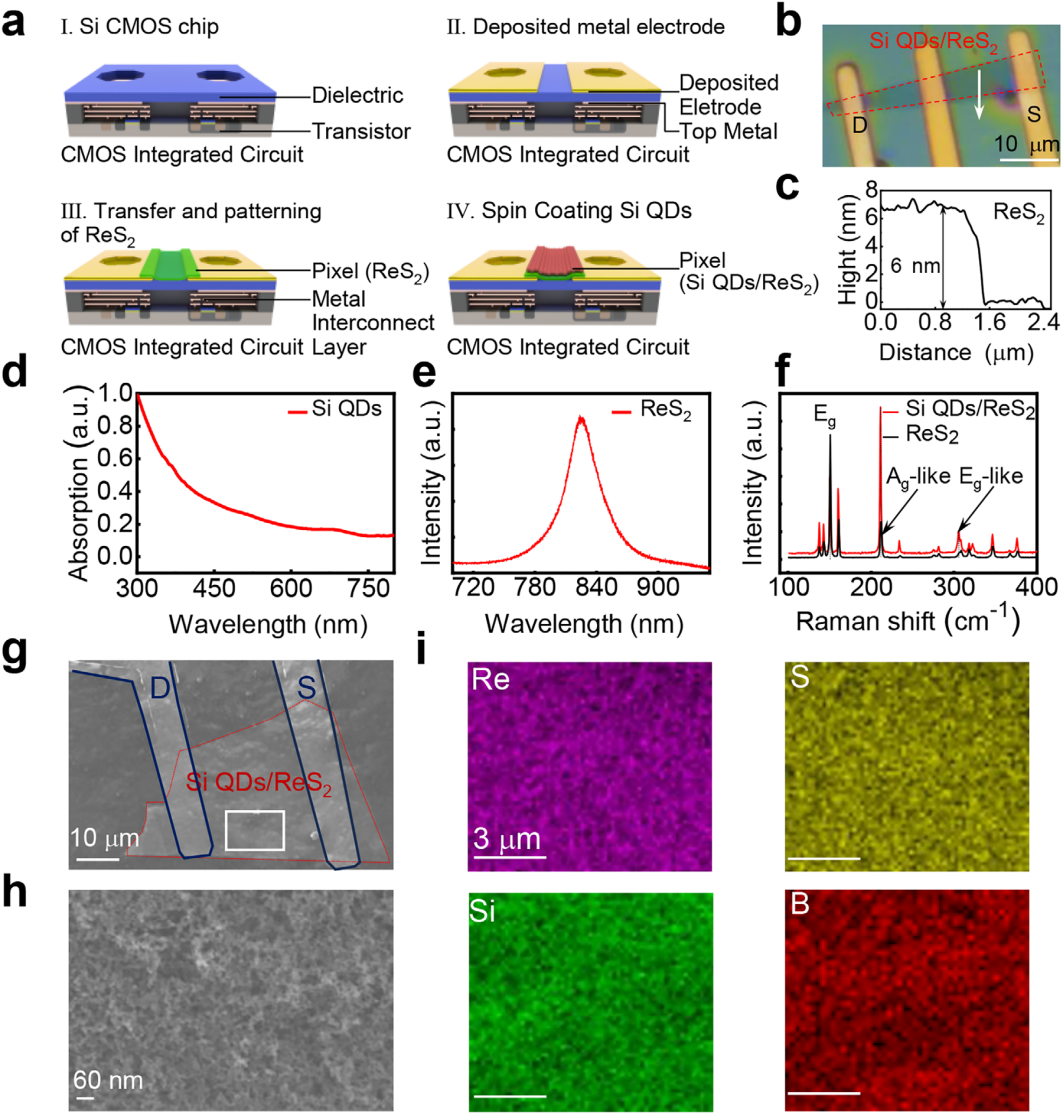
Fabrication process flow for SiQDs/ReS_2_ neuromorphic image sensor heterogeneous chip. (a) Preparation process of the SiQDs/ReS_2_ neuromorphic image sensor heterogeneous chip. (b) Optical microscope image of SiQDs/ReS_2_ phototransistor. (c) Height distribution along the white curve in Figure 2b (Before spin‐coating Si QDs) as observed by atomic force microscopy. The atomic force microscopy (AFM) scan clearly shows the thickness of ReS_2_ with a step height of 6 nm. (d) UV–vis absorption spectrum of the SiQDs. (e) The steady‐state PL spectrum of ReS_2_, measured under 532 nm excitation, features a peak at approximately 1.525 eV. (f) Raman spectrum of ReS_2_ before and after spin‐coating with SiQDs. (g) The top‐view scanning electron microscope image of Si QDs/ReS_2_ phototransistor. Scale bar: 10 µm. (h) The top‐view scanning electron microscope image of a localized region on the surface of the Si QDs/ReS_2_ heterojunction. Scale bar: 60 nm. (i) EDS mapping of the location indicated by the rectangular box in Figure 2g.

Figure 2a shows a schematic diagram of the heterogeneous integration process of the SiQDs/ReS_2_ NISH chip. (I) CMOS chips were fabricated at a commercial foundry using a standard 180 nm silicon process. (II) Metal electrodes are subsequently defined on the prepared chips by photolithography, resist development, and sputter deposition. (III) CVD ReS_2_ is transferred onto the chip with the prepared metal electrode through the wet transfer process. The sample is then baked to remove residual moisture and to promote intimate adhesion and reliable electrical contact. Following this, the ReS_2_ layer is patterned to define the desired device structure. (IV) Si QDs dispersed in ethanol are spin‐coated onto the chip with pre‐patterned ReS_2_. The Si QDs/ReS_2_ phototransistors are stacked on the chip surface and achieve vertical interconnection with the underlying CMOS circuits through metal interconnect devices. The Si QDs/ReS_2_ phototransistors convert optical signals into electrical signals, which are then transmitted to the underlying CMOS integrated circuit for further processing via metal interconnects. Figure 2b shows an optical micrograph of the SiQDs/ReS_2_ phototransistor. As shown in Figure 2c, the thickness of the ReS_2_ nanosheet measured along the white reference line in Figure 2b using atomic force microscopy (AFM) is 6 nm. As shown in Figure 2d, the SiQDs exhibit strong absorption across the ultraviolet to visible spectral range. The photoluminescence spectrum of ReS_2_ (Figure 2e) displays a peak at 813 nm, consistent with an optical bandgap of 1.525 eV. Following spin‐coating of the SiQDs, the characteristic Raman modes of ReS_2_ redshift relative to the pristine flake (Figure 2f). Figure 2g presents a top‐view scanning electron micrograph (SEM) of the Si QDs/ReS_2_ heterojunction phototransistor. The magnified SEM image in Figure 2h, taken from the boxed region in Figure 2g, reveals Si quantum dots attached to the ReS2 surface. The energy‐dispersive X‐ray spectroscopy (EDS) spectrum in Figure S1, acquired at the marked site in Figure 2g, identifies Re, S, Si, and B. The corresponding EDS elemental maps (Figure 2i) further confirm the spatial distribution of these elements across the heterojunction.

### 2.3 Electric Performance of SiQDs/ReS_2_ Phototransistor

**FIGURE 3:**
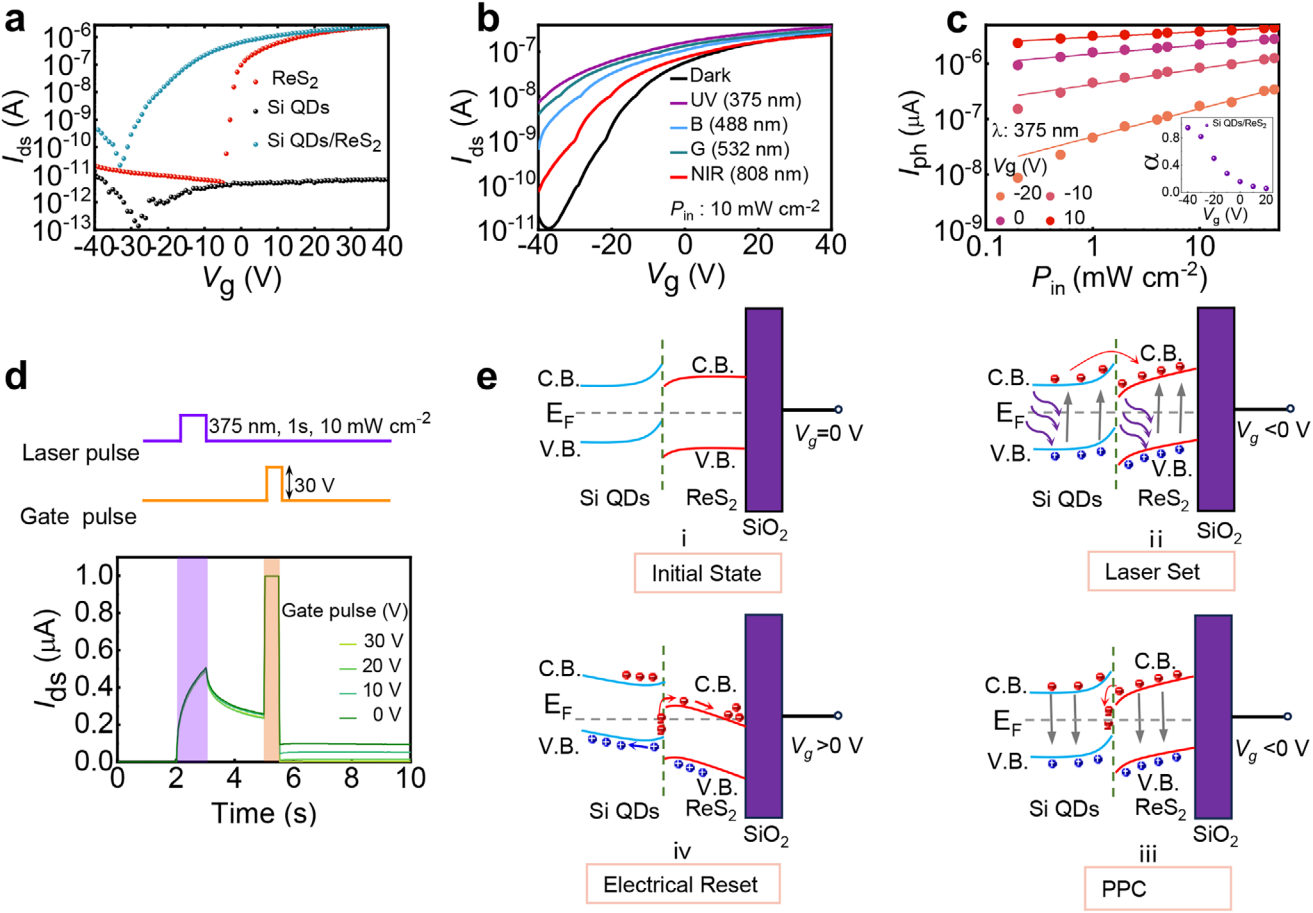
Electrical properties of Si QDs/ReS_2_ phototransistor. (a) Transfer curves of Si QDs, ReS_2_, and Si QDs/ReS_2_ transistors. (b) Transfer characteristic curves of the Si QDs/ReS_2_ artificial photoreceptor at different wavelengths and the same light intensity. (c) Photocurrent versus laser power for different back‐gate voltages. The embedded graph shows the α. (d) The transient characteristic of Si QDs/ReS_2_ artificial synaptic photoreceptor operated with alternating light and electrical pulses. (e) Energy band mechanism diagram of the PPC behavior of Si QDs/ReS_2_ artificial synaptic photoreceptor at different gate voltages.

The transfer characteristics of the ReS_2_ transistor before and after spin coating the Si QDs layer are shown in Figure 3a. The threshold voltage (Vth) of the SiQDs/ReS_2_ transfer curve exhibits a negative offset, indicating electron transfer from SiQDs to ReS_2_ (Figure S2). Owing to quantum confinement and the large inter‑dot energy barriers, the SiQDs film provides effective lateral self‑isolation, obviating additional isolation steps (Figure 3a). The transfer characteristic curve of SiQDs/ReS_2_ under different wavelengths is shown in Figure 3b. Under the same power intensity (*P*_in_), SiQDs/ReS_2_ demonstrate a significantly stronger response to ultraviolet light compared to other wavelengths (Figure 3b). The transfer (*I*_ds_‐*V*_g_) of SiQDs/ReS_2_ phototransistor under 488 nm (blue), 532 nm (green), and 808 nm (near‐infrared) with different incident power (Figure S3). The relationship between the photocurrent (*I*_Ph_) and light intensity (*P*_in_) of the Si QDs/ReS_2_ at 375 nm for different back‐gate voltages is shown in Figure 3c, demonstrating that the photocurrent is generated by the photogating effect, following the relation, *I*_Ph_∝*P*_in_^𝑎^ 𝑤𝑖𝑡ℎ α<1 [46].

To further elucidate the operating mechanisms of the Si QDs/ReS_2_ artificial synaptic photoreceptor under simultaneous gate modulation and pulsed optical excitation, we performed time‐resolved photoresponse, revealing pronounced persistent photoconductivity (PPC). Figure 3d summarizes the synaptic response of the Si QDs/ReS_2_ photoreceptor under interleaved optical and electrical stimuli. Upon illumination, the channel current increases abruptly. After the light is switched off, it relaxes slowly toward a new quasi‐steady state rather than immediately returning to the dark level. A subsequent positive gate‐voltage pulse efficiently erases the residual conductance, restoring the current to the dark state. The corresponding band diagram level operating mechanism during periodic optical excitation and electrical resetting is schematically illustrated in Figure 3e. In the initial state (Stage i), the Si QDs/ReS_2_ heterojunction is at equilibrium under zero gate bias (*V*_g_ = 0), and no net interfacial charge transfer occurs. Applying a constant negative gate voltage (*V*_g_) induces band bending at the interface. Under optical excitation, photogenerated carriers are captured by interfacial defect states, and their slow release produces persistent photoconductivity (PPC) (Stages ii and iii). In the reset stage (Stage iv), applying a positive *V*_g_ pulse lowers the interfacial barrier and accelerates the detrapping of the captured electrons, thereby suppressing PPC and restoring the dark current to its baseline value. The response of the Si QDs/ReS_2_ artificial photonic synapse under alternating optical excitation and electrical reset (Figure S7).

### 2.4 Static Image Acquisition and Full‐Color Image Processing

**FIGURE 4:**
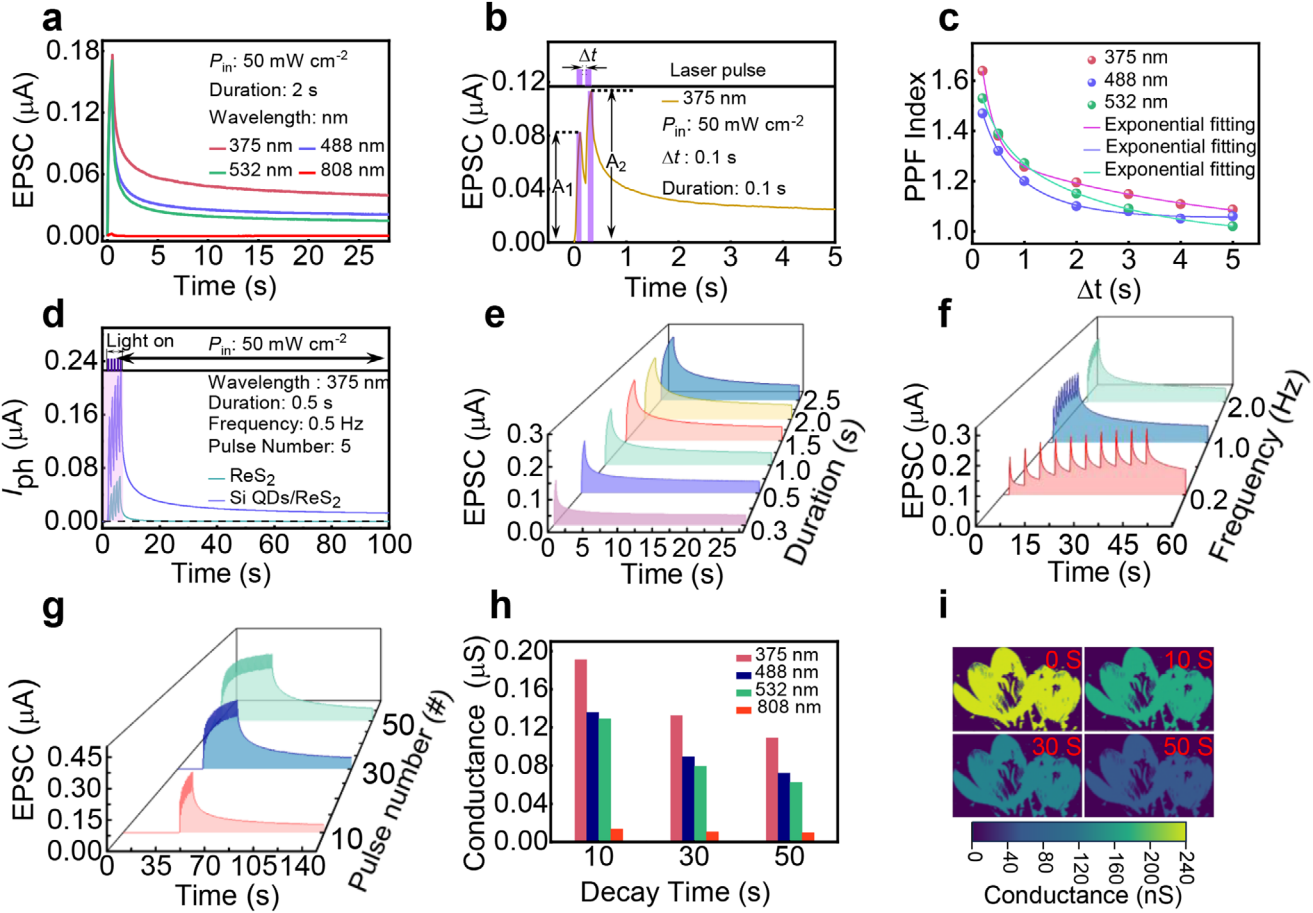
Synaptic performance of Si QDs/ReS_2_ photoreceptor. (a) EPSC of Si QDs/ReS_2_ artificial synaptic photoreceptor induced by laser pulses at different wavelengths. (b) EPSC of the Si QDs/ReS_2_ artificial synaptic photoreceptor induced by two successive laser pulses with an interval time (Δt) of 100 ms. (c) Dependence of the PPF index on Δt for the laser spikes at different wavelengths. (d) Time‐dependent photoresponse of ReS_2_ and Si QDs/ReS_2_ under 375 nm pulsed illumination. (e–g) EPSC of the Si QDs/ReS_2_ artificial synaptic photoreceptor induced by a 375 nm laser pulse with different pulse duration, frequency, and pulse number. (h) Statistics of channel conductance versus decay time measured after the termination of 50 optical pulses at 375 nm (UV), 488 nm (blue), 532 nm (green), and 808 nm (near‐infrared). (i) The difference between the purple stamens and red flowers as the decay time of light pulses increases (the original image used in panel (i), “ Crocus, Purple, Flower wallpaper image,” licensed under CC0 License via Pixabay.com).

Figure 4a presents the excitatory postsynaptic current (EPSC) of Si QDs/ReS_2_ artificial synaptic photoreceptor excited by light pulses of different wavelengths. The device exhibits distinct wavelength dependence, with high sensitivity to ultraviolet illumination and reduced sensitivity to near‐infrared light. This reflects a wavelength‐selective strategy similar in principle to the color‐selective vision of bees, designed to efficiently extract salient spectral information. The paired‐pulse facilitation (PPF) of Si QDs/ReS_2_ artificial synaptic photoreceptor is shown in Figure 4b, where two successive 375 nm light pulses of 500 ms duration with different intervals at a *V*_g_ value of ‐20 V trigger the PPF response of Si QDs/ReS_2_ artificial synaptic photoreceptor. The significantly stronger second peak (*A*_2_) over the first peak (*A*_1_) in EPSC mirrors biological PPF behavior. The PPF index, defined as *A*_2_/*A*_1_, decreases monotonically with the inter‐pulse interval Δt, as shown in Figure 4c. As interval time increases from 0.2 to 5 s, the modulation ratio steadily decreases from 163% to 108%. This ratio can be accurately modeled by a double‐exponential function:

$$y=A_{0}+A_{1}\times \exp\left(-\frac{\Delta t}{\tau_{1}}\right)+A_{2}\times \exp\left(-\frac{\Delta t}{\tau_{2}}\right)$$

where *A*_1_ and *A*_2_ are facilitation constants, and Δ*t*, *τ*_1_ and *τ*_2_ represent the interval time in two pulses, which are relaxation time, fast and slow relaxation time, respectively. The black line is a fitted curve that matches the experimental data. The two characteristic timescales, *τ*_1_ and *τ*_2_ are estimated to be 0.25 and 4.60 s, respectively. *τ*_1_ is an order of magnitude larger than *τ*_2_, indicating a reasonable fit.

Figure 4d demonstrates the photomemory retention capability of the pristine ReS_2_ and Si QDs/ReS_2_ heterojunction under optical pulse excitation. After 10 s of light extinction, the photocurrent retention rate of the pristine ReS_2_ device decayed to 0%, while the Si QDs/ReS_2_ device maintained over 25% of its initial photoconductive state. The extended persistence is detailed in Figure S4 and is primarily attributed to surface defect states in the Si QDs, which act as traps for photogenerated holes once illumination ceases, thereby suppressing recombination and prolonging carrier lifetimes [45]. As shown in Figure 4e–g and Figure S5, increasing pulse width, frequency, and pulse number produces higher EPSC amplitudes and longer retention times, enabling a controlled transition between short‐term potentiation (STP) and long‐term potentiation (LTP).

The postsynaptic current increases with the number of laser spikes (from 10 to 50), indicating synaptic potentiation and enhanced visual discrimination under repeated optical training. Figure 4h shows the conductance of Si QDs/ReS_2_ artificial synaptic photoreceptor at different wavelengths after 50 consecutive light pulses as a function of decay time. Based on the change in decay time for different conductance states, the corresponding output images appear as shown in Figure 4i and Figure S5f, where the flower pattern is extracted by sampling the UV and red components of the initial color image through the synaptic device. In this mapping process, the violet and red colors of the flowers are mapped to the conductance values of the device after excitation by light pulses at wavelengths of 375 and 808 nm, respectively, presenting the corresponding output images according to the change in decay time of the different conductance states, and maintaining the violet feature even when the decay time is increased to more than 50 s. The simulation results indicate that Si QDs/ReS_2_ artificial synaptic photoreceptor can effectively distinguish between purple and red features without the need for additional filters, and exhibit a significant memory effect for purple. This demonstrates that the device can selectively extract information at different wavelengths, similar to the color‐selective vision of bees, enabling the efficient extraction of key information [42, 43].

### 2.5 Si QDs/ReS_2_ Artificial Synaptic Photoreceptor Operation for Motion Recognition

**FIGURE 5:**
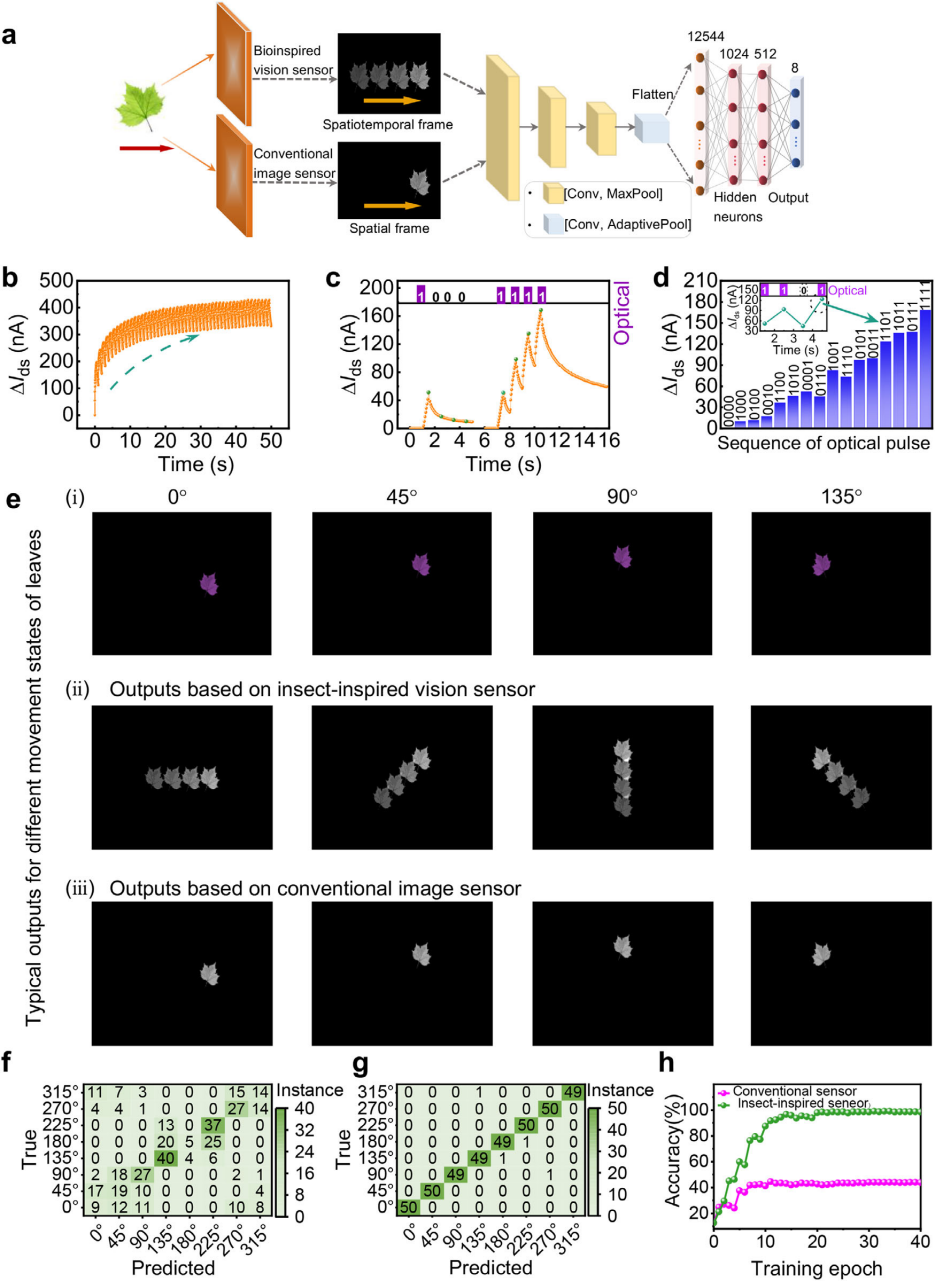
Action recognition based on Si QDs/ReS_2_ insect‐inspired visual sensors and traditional image sensors. (a) Schematic diagram of a neural network for leaf motion trajectory recognition based on insect‐inspired vision and traditional image sensors. The insect‐inspired vision sensor outputs spatiotemporal fusion feature frames, whereas traditional image sensors only output discrete spatial frames. Time‐dependent drain current (Δ*I*_ds_) in response to light pulse stimulation: (b) a 2 Hz optical pulse train; (c) a single light pulse (0.5 s on) and four consecutive pulses (0.5 s on, 0.5 s off); and d) a four‐bit optical pulse sequence, where ‘1’ and ‘0’ denote the on and off of a light pulse at *V*_g_ = −20 V, respectively. *V*_ds_ = 1 V for all measurements. (e) Subpanel (i) shows leaves moving in the image plane along four directions (0°, 45°, 90°, 135°); (ii) and (iii) present the corresponding outputs from the insect‐inspired visual sensor and a conventional image sensor, respectively. Confusion matrices for direction‐of‐motion classification of eight leaf types using (f) a conventional image sensor and g) an insect‐inspired vision sensor. (h) Action recognition accuracy of the insect‐inspired visual sensor compared with that of the conventional image sensor.

Insects possess far greater sensitivity to moving objects than humans, owing to the unique non‐electrical pulse‐stimulated hierarchical neuron structure within their compound eye visual system [14]. The information transmission rate and coding efficiency of these neurons significantly surpass those of pulse neurons in the human visual system. Through their miniature yet sophisticated visual systems, insects can efficiently perceive dynamic scenes. This biological mechanism provides crucial insights for developing bio‐inspired optoelectronic neuromorphic devices that mimic insect vision. We constructed a dataset containing blade motion trajectories across eight angular orientations (0°, 45°, 90°, 135°, 180°, 225°, 270°, and 315°) for training and testing this vision system. The sensor array feeds the acquired image data into the neural network architecture illustrated in Figure 5a. This network comprises a four‐stage convolutional backbone for feature extraction and a three‐layer fully connected classification head, ultimately performing an eight‐class classification task. Inspired by biological vision, this sensor mimics the information processing mechanism of the bee retina to integrate spatiotemporal information from consecutive frames into a single compressed representation. In contrast, traditional image sensors retain only spatial information within a single frame.

A bioinspired photonic neuromorphic array based on Si QDs/ReS_2_ phototransistors emulates the bee visual system's direction‐selective perception of leaf motion across eight predefined azimuths. Under repetitive pulsed illumination at 375 nm (50 mW cm^−2^, 100 Hz), the device exhibits cumulative photocurrent potentiation followed by saturation, exhibiting response characteristics similar to insect hierarchical neurons (Figure 5b). These neurons efficiently integrate and encode pulse trains from photoreceptors through temporal summation, thereby extracting the spatiotemporal features of moving objects without an explicit refractory constraint. To further assess the device's capacity for temporal encoding, we applied two distinct four‐pulse optical sequences in a separate protocol, each with a pulse width of 0.5 s and an inter‐pulse interval of 0.5 s, the final steady‐state response currents (Δ*I*_ds_) exhibited distinct differences (Figure 5c), indicating temporal summation characteristics. The inset in Figure 5d illustrates the encoding process for the device code “1101,” where the device response current (Δ*I*_ds_) exhibits gradient changes at four distinct time points. The digits ‘1’ and “0” represent illumination conditions of 50 mW cm^−^^2^ and darkness, respectively. The main panel of Figure 5d presents Δ*I*_ds_ of the Si QDs/ReS_2_ heterojunction phototransistor synapse for all 16 possible four‐pulse sequences, each with a pulse width and inter‐pulse interval of 0.5 s. The synaptic output is governed primarily by the temporal order and spacing of the light pulses. These results demonstrate robust temporal optical encoding in the device, analogous to the hierarchical processing of dynamic visual information in biological neural systems.

Each sample in the dataset maintains a fixed image resolution of 640 × 512 pixels, encompassing four motion directions (0°, 45°, 90°, and 135°) as illustrated in Figure 5e(i), while the remaining directions (180°, 225°, 270°, 315°) are shown in Figure S8. The dataset consists of 608 training samples and 400 test samples. To increase diversity and improve model robustness, we introduced random variations in step length (stride), slight path curvature, and jitter orthogonal to the motion direction during data synthesis, applied across inter‐frame displacements, trajectory orientations, and spatial positions. The biologically‐inspired sensor outputs temporal‐state fused compressed frames (Figure 5e(ii)), which integrate spatiotemporal information from continuous motion sequences. This integration renders discernible trajectory contours, thereby providing richer feature representations for motion recognition. Conversely, conventional image sensors capture only static stimuli within individual frames (Figure 5e(iii)), inherently incapable of encoding temporal relationships in motion dynamics.

To quantify motion direction recognition, an eight‐class confusion matrix was constructed (Figure 5f,g). Matrix analysis reveals that the model trained on conventional single‐frame static datasets exhibits dispersed off‐diagonal distributions, indicating significant inter‐class confusion. In contrast, the model trained with temporal‐state fused compressed frames demonstrates concentrated diagonal distributions, reflecting well‐defined classification boundaries with minimal inter‐class ambiguity. Figure 5h comparatively presents the training accuracy curves of bio‐inspired versus conventional vision sensors. After 40 training epochs, the bio‐inspired sensor‐based model achieves a final motion accuracy of 97.58% in the test. The conventional sensor‐based model attained merely 49.59%. The performance superiority of the bio‐inspired sensor fundamentally stems from its compressed‐frame representation, which integrates complete spatiotemporal signatures of motion dynamics. This enables the model to capture continuous spatiotemporal features, transcending the limitations of static single‐frame information dependency.

### 2.6 Depth Perception by Si QDs/ReS_2_ NISH Chip

**FIGURE 6:**
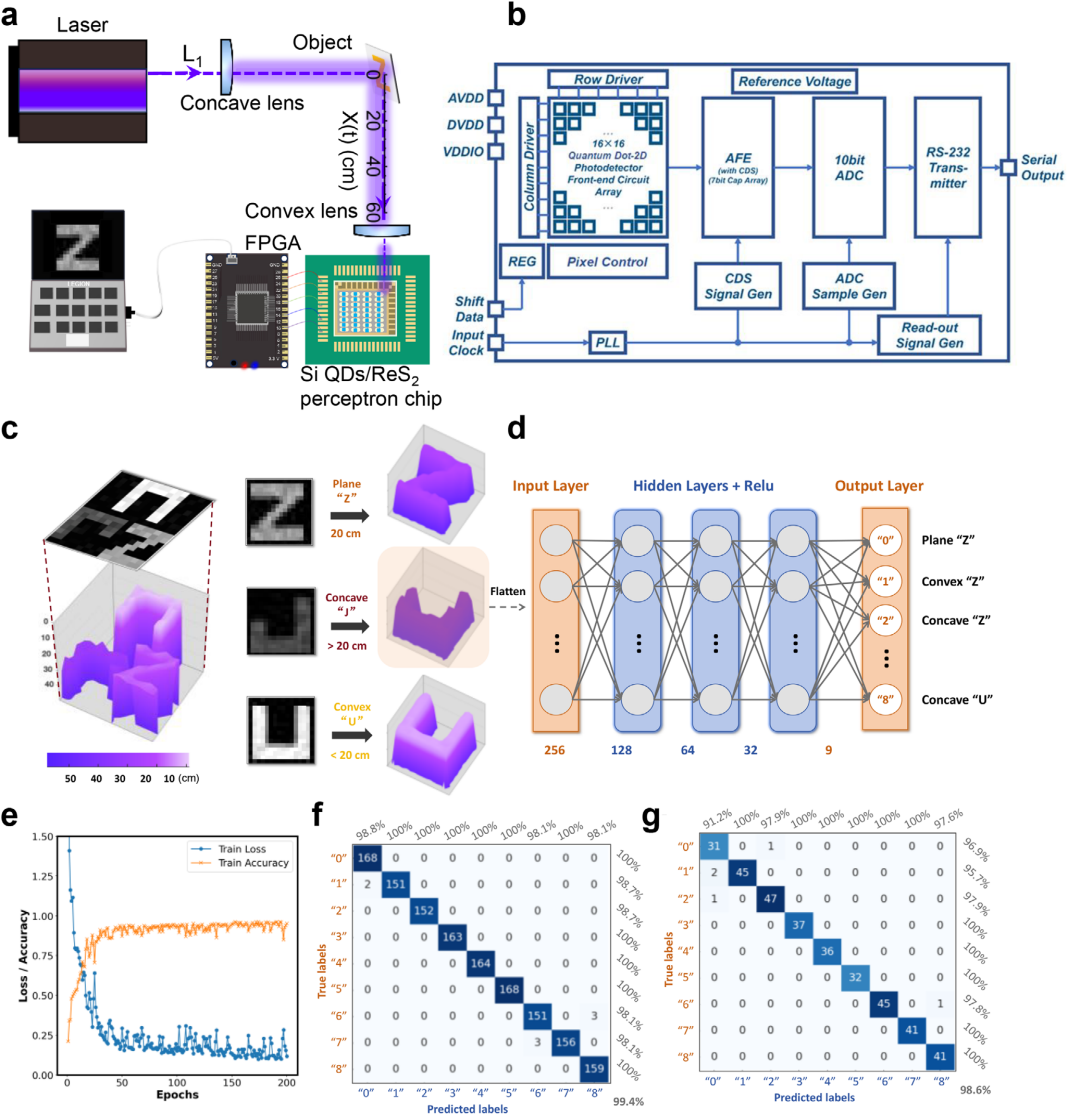
Depth imaging and classification in a fully‐connected neural network architecture. (a) The imaging system setup for depth detection of the Si QDs/ReS_2_ neuromorphic image sensor chip. (b) Architecture block diagram of Si QDs/ReS_2_ neuromorphic image sensor heterogeneous chip. (c) Schematic representation of depth perception. Three objects in different shapes of “Z”, “J”, and “U” are pieced together with different depths, which are calculated by the EPSC of devices. d) The objects of “Z”, “J”, and “U” in the resolution of 8 × 8 are also classified into “Plane”, “Concave”, and “Convex” with their distance thresholds relative to a reference plane (20 cm), which are applied to a 9‐category classification. Neural network architecture. A fully connected network with an input layer (mapped to depths), three hidden layers with activation, and an output layer for 9‐category classification. (e) Training dynamics. Loss and accuracy statistics over 200 epochs, indicating rapid convergence (loss approaching 0.1) and high training accuracy (> 98%). (f,g) Confusion matrix in both train set and test set, achieving 99.4% accuracy on the train set and 98.6% on the test set, confirming the robustness of the classification model.

Extensive efforts have been devoted to biomimetic compound‐eye systems, including hemispherical photodetector arrays [47, 48], curved microlens arrays [49], and ommatidium‐inspired imaging architectures [50, 51]. These structural approaches have achieved wide fields of view [47, 48, 50, 52], imaging capability [53], motion detection [51, 54], and compact form factors. Nevertheless, they often require complex fabrication on curved or flexible substrates, which complicates integration with conventional planar CMOS technology. Inspired by the parallel architecture of insect vision, in which numerous ommatidia capture local visual cues and relay them through parallel neural pathways [42], we developed a Si QDs/ReS_2_ artificial synaptic photoreceptor array heterogeneously integrated on a standard 180‐nm CMOS chip. Although the array adopts a planar 16×16 layout rather than the curved geometry of a natural compound eye, it effectively emulates ommatidial parallel processing. Figure 6b illustrates all modules of the Si QDs/ReS_2_ NISH chip readout circuit and the signal transmission process between them. Details regarding the functions of each module within the chip and the signal acquisition and readout process within the circuit are shown in Figure S9 and Note S2.

To preliminarily evaluate the chip's spatial sensing, we performed a hardware demonstration using a simplified static mapping based on distance‐dependent light attenuation. Figure 6a gives a schematic representation of the depth perception where the 16 × 16 pixel array can induce unique EPSC patterns, enabling depth mapping through a calibrated relationship between EPSC values and depths in centimeters (The calibration and mapping mechanism are detailed in Note S3, Figures S10, and S11). According to the distinct EPSC responses of objects positioned at varying depths relative to a reference plane (in depth of 20 cm), 9 object categories were designed, including plane “Z”, concave “Z”, convex “Z”, plane “J”, concave “J”, convex “J”, plane “U”, concave “U” and convex “U”, where concave objects is classified with depths over 20 cm and convex objects with depths under 20 cm. The EPSC images and the mapped depth image of plane “Z”, concave “J”, and convex “U” are shown in Figure 6c. Obviously, the convex image shows a brighter EPSC image because it is closer to the device plane. A synthetic dataset was constructed by randomly applying EPSC values to the character image in the corresponding depth type, and each category contains 200 images. Figure 6d describes the applied neural network architecture. A fully connected neural network was designed to classify the 9 categories, which comprises an input layer (mapped from EPSC values to depth information), three hidden layers (neurons of 128, 64, and 32 relatively) with activation function of ReLU to enhance nonlinear feature extraction, and an output layer. The training statistics in Figure 6e demonstrate rapid convergence, and after 50 epochs, the loss decreases to 0.25, and training accuracy exceeds 98%. Finally, the model achieved 99.4% train accuracy and 98.6% test accuracy, underscoring its robustness. Figure 6f,g gives the confusion matrices, revealing near‐perfect classification across all classes, with minimal mislabeling between geometrically similar categories. This approach integrates bio‐inspired detector arrays with data‐driven learning, offering a scalable solution for depth‐aware 3D sensing.

## 3 Conclusions

In summary, we designed and fabricated a Si QDs/ReS_2_ artificial synaptic photoreceptor that emulates the spectral selectivity and temporal information processing of the insect visual system. Under ultraviolet excitation, the device exhibits strong photoresponse and sustained photoconductive behavior. This device implements fundamental synaptic behaviors such as PPF, STP, and LTP. It also possesses optical pulse sequence encoding capabilities, enabling efficient processing of temporal light information to simulate the dynamic visual encoding mechanism achieved by layered neurons in insects. Through heterogeneous integration technology, the Si QDs/ReS_2_ artificial synaptic photoreceptor was monolithically integrated with CMOS readout circuits, enabling 3D visual perception capabilities. Utilizing postsynaptic current mapping, the chip achieves object depth classification and recognizes nine geometric shapes with 99.4% accuracy. This research provides a feasible technical pathway from materials and devices to system integration, laying a crucial foundation for developing next‐generation neuromorphic vision chips with low power consumption, high real‐time performance, and multimodal perception. It holds broad application prospects in autonomous driving, robotic vision, and edge intelligence.

## 4 Experimental Section

### 4.1 Si QDs/ReS_2_ Phototransistor Fabrication

Few‐layer ReS_2_ flakes were mechanically exfoliated from bulk single crystals (Shenzhen Six Carbon, China) using adhesive tape and transferred onto heavily doped p‐type Si substrates with 285 nm SiO2 using a polydimethylsiloxane (PDMS) stamp. Subsequently, Cr/Au contacts (5/30 nm) were patterned by direct laser writing and metallized via thermal evaporation to serve as the source and drain electrodes. Finally, transfer the ethanol suspension containing Si QDs nanoparticles to the prepared ReS_2_ substrate with source and drain electrodes using a pipette. Then, spin‐coat the suspension evenly using a spin coater at a speed of 1000 rpm for 1 min. Finally, dry the prepared Si QDs/ReS_2_ heterojunction crystals in a vacuum environment at 100°C for 30 min.

### 4.2 Si QDs/ReS_2_ Neuromorphic Image Sensor Chip Fabrication

Monolayer ReS_2_ (Shenzhen Six Carbon, China) was transferred onto a pre‐fabricated CMOS readout integrated circuit (ROIC) via a wet‐transfer process. A polymethyl methacrylate (PMMA, 950k A4) support layer was spin‐coated on the ReS_2_ and soft‐baked at 120°C for 90 s. The substrate is transferred to a pre‐fabricated CMOS readout circuit chip using deionized water. The assembly was dried in a vacuum oven for 12 h and subsequently baked at 100°C for 30 min on a hot plate. The PMMA was removed by immersion in acetone at 50°C for 30 min, followed by rinsing with isopropyl alcohol. The ReS_2_ was patterned by UV photolithography (MA6‐BSA) to define pixel channels that made full contact with the two electrodes of each pixel. Argon plasma etching (PT‑5SM) was employed to remove ReS_2_ outside the defined pixel regions, thereby ensuring electrical isolation between adjacent pixels. Finally, an ethanol suspension of Si quantum dots (Si QDs) was dispensed onto the chip and spin‐coated at 1000 rpm for 60 s to achieve uniform coverage.

The device was then vacuum‐dried at 100°C for 30 min, completing the fabrication of the Si QDs/ReS_2_ neuromorphic image sensor.

### 4.3 Material Characterization

Raman spectra were acquired using a commercial spectrometer (RM 2000) with 532 nm excitation in the backscattering geometry. ReS_2_ thickness was determined by AFM (MFP‐3D Origin+, Oxford Instruments). The surface morphology was examined using a field‐emission scanning electron microscope (FE‐SEM, Sigma 300, Zeiss, Oberkochen, Germany).

### 4.4 Electrical and Optical Measurements

Electrical characterization was carried out on a probe station using a Keithley 4200 semiconductor parameter analyzer. The device's optoelectronic characteristics were measured using an Mstarter 200 high‐precision photocurrent scanning microscope (Nanjing Metatest Optoelectronics Co., Ltd.) in combination with MGL‑III laser sources (200 mW) operating at 375, 488, 532, and 808 nm. Optical power was measured using Thorlabs S130VC optical power meters. All measurements were performed under well‑shielded conditions to minimize environmental interference.

### 4.5 Image Processing With Si QDs/ReS_2_ Photodetector

The original image used in Figure 4(i) was retrieved from Pixabay (https://pixabay.com/photos/crocus‐purple‐flowers‐purple‐petals‐249359/).

## Conflicts of Interest

The authors declare no conflicts of interest.

## Supporting information




**Supporting File**: advs75388‐sup‐0001‐SuppMat.pdf.

## Data Availability

The data that support the findings of this study are available from the corresponding author upon reasonable request.
