# Supplementary material for: CMOS‐Integrated Synaptic Photoreceptor Chip Inspired by Insect Visual Processing
Source: Adv Sci (Weinh). 2026 Apr 21;13(40):e75388. doi: 10.1002/advs.75388 (PMC13335448; doi:10.1002/advs.75388)
Supplement: Supplementary file 1 — Supporting File: advs75388‐sup‐0001‐SuppMat.pdf. [file ADVS-13-e75388-s001.pdf]

Supporting Information

CMOS-Integrated Synaptic Photoreceptor Chip Inspired by Insect Visual Processing

Jian Chai[1#], Xinyi Xu[1#], Yue Wang[4#], Xiaochen Wang[1], Hailiang Wang[1], Yunfei Xie[1], Xinwei Zhang[1], Haoyu Wang[1], Hao Ning[1], Jiangming Lin[1], Yongliang Xie[1], Qihai Jiang[3], Baoshi Qiao[3], Xiaolei Ding[3], Luyao Ma[2], Shukai Duan[2], Zhenyi Ni[4], Huan Hu[3], Xin He[5,7], Fei Xue[5], Feichi Zhou[6], Lingfei Li[1*], Srikrishna Chanakya Bodepudi[1*], Rui Yuan[2*], Bin Yu[1*], Yang Xu[1*]*

[1]College of Integrated Circuits, State Key Laboratory of Silicon and Advanced Semiconductor Materials, ZJU-HIC, Center of CMOS IC Manufacturing Process and Design, Zhejiang University, Hangzhou 310027, China.

[2]College of Artificial Intelligence, Southwest University, Chongqing 400715, China

[3]ZJU-UIUC Institute, International Campus, Zhejiang University, Haining 314400, China

[4]State Key Laboratory of Silicon and Advanced Semiconductor Materials & School of Materials Science and Engineering, Zhejiang University, Hangzhou, Zhejiang 310027, China

[5]ZJU-Hangzhou Global Scientific and Technological Innovation Center, Zhejiang University, Hangzhou 311215, China.

[6]School of Microelectronics, Southern University of Science and Technology, Shenzhen, 518000, China

[7]Center for Quantum Matter, School of Physics, Zhejiang University, Hangzhou 310058, China

[#]These authors contributed equally to this work.

*Corresponding author:
Email: yangxu-isee@zju.edu.cn; yuanruiswu@swu.edu.cn; yu-bin@zju.edu.cn; lingfei_li@zju.edu.cn; bodepudi@zju.edu.cn; xiaochenwang@zju.edu.cn;

EDS spectrum of Si QDs/ReS2

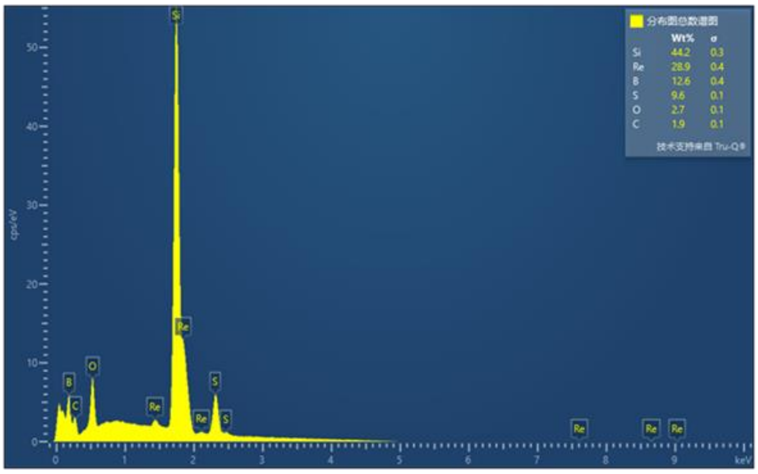

Figure S1: The EDS spectrum of Si QDs/ReS_2 phototransistor detected from the marked positions in Figure 2g.

Optoelectronic performance of Si QDs/ReS2

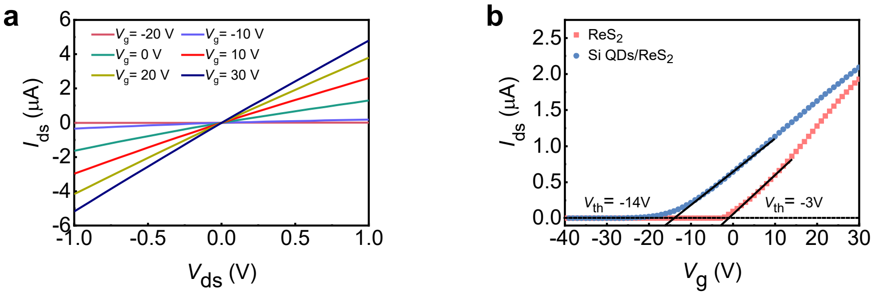

Figure S2. a) Output characteristics (I_{ds}–V_{ds}) of the Si QDs/ReS_2 transistor, demonstrating Ohmic contact between ReS_2 and Au electrodes. b) Transfer characteristics of ReS_2 field-effect transistors before and after spin-coating Si QDs.

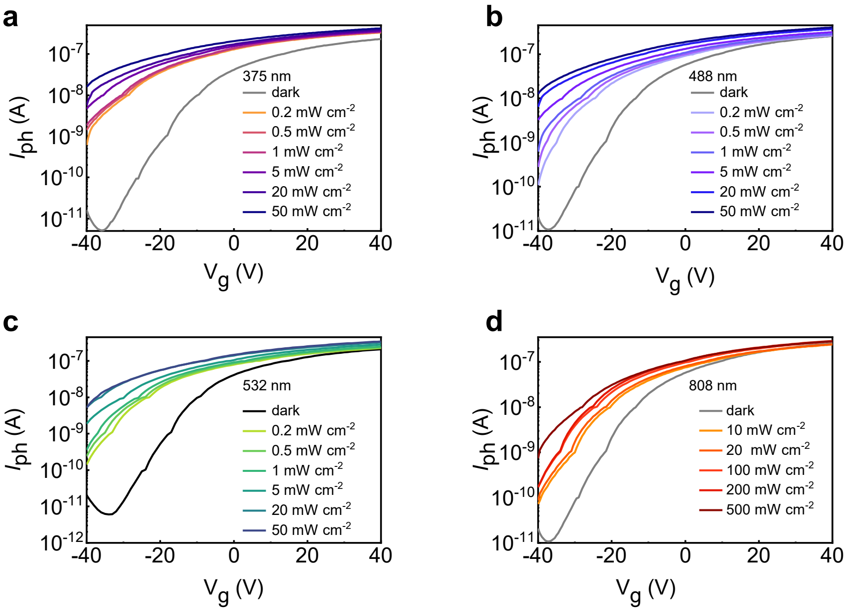

Figure S3. Transfer characteristics of Si QDs/ReS_2 at V_{ds}= 0.1V at dark and different wavelength light-irradiation conditions: a) 375 nm. b) 488 nm. c) 532 nm. d) 808 nm.

Figure S2 (a) shows the output characteristics (I_{ds}–V_{ds}) of the Si QDs/ReS_2 phototransistor. The nearly linear dependence of I_{ds} on V_{ds} confirms the formation of good ohmic contacts between the Au electrodes and the ReS_2 channel. Figure S2 (b) displays the transfer characteristic curves of ReS_2 before spin-coating Si QDs and of Si QDs/ReS_2 after spin-coating Si QDs. ReS_2 exhibits n-type transport behavior, and the threshold voltage of the device shifts negatively with increasing gate voltage after Si QD spin-coating, indicating electron transfer from Si QDs to ReS_2. Figure S3 (a-d) respectively shows the I_{ds}-V_{gs} curves under different wavelengths and light power densities, indicating that the device exhibits sensitive photo-response.

Synaptic properties of Si QDs/ReS2

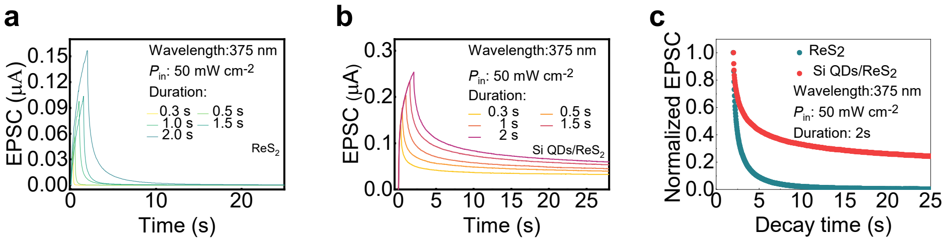

Figure S4. EPSC responses of a) ReS_2 and b) Si QDs/ReS_2 under 375 nm light pulse with different durations. c) Normalized channel conductance decay of ReS_2 and Si QDs/ReS_2 following optical pulse stimulation with a pulse width of 2 s. After 10 seconds of light exposure cessation, the normalized EPSC of the pristine ReS_2 device and the Si Si QDs/ReS_2 device were 0 and 0.25, respectively.

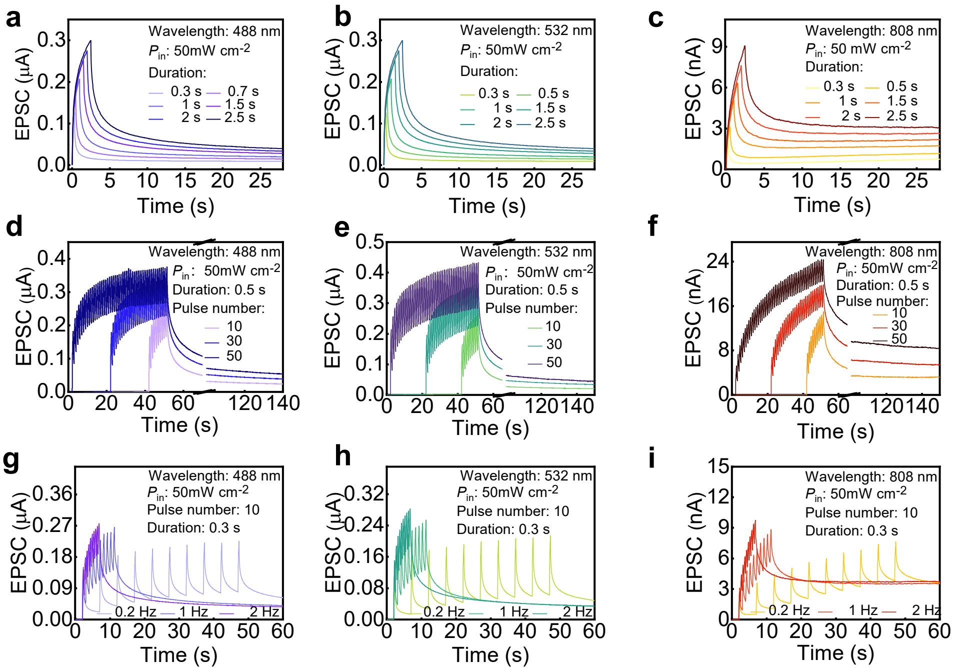

Figure S5. The Si QDs/ReS_2 response to various light pulse duration time a-c), numbers d-f), and frequency g-i). The light source in a), d), and g) is at 488 nm; in b), e), and h) is at 532 nm; and in c), f), and i) is at 808 nm. V_d and V_g are 1V and -20V, respectively.

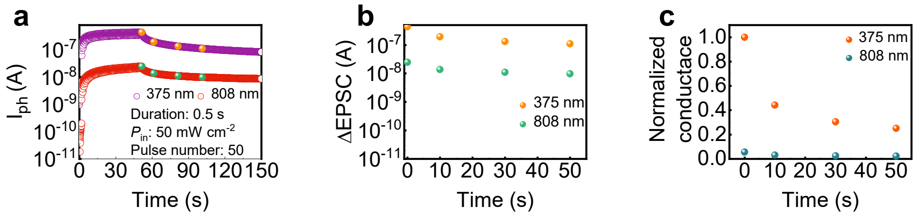

Figure S6. a) Photonic synapse characteristics of Si QDs/ReS_2 phototransistors after 50 pulses (corresponding UV and red light, respectively), with a pulse frequency of 2 Hz

and light intensity of 50 mW cm^{-2}. b) ΔEPSC values measured at different decay time points for the device after 50 light pulses at wavelengths of 375 nm (UV light) and 808 nm (red light). c) Normalized conductance values measured at different decay time points for the device after 50 light pulses at wavelengths of 375 nm (UV light) and 808 nm (red light). The calculation methods for ΔEPSC and normalized conductance are detailed in Supplementary Note 1.

Fig. S4 (a) and (b) show the excitatory postsynaptic current (EPSC) responses measured in ReS_2 devices before and after spin-coating Si QDs, respectively, under different light pulse width stimuli. It is evident that under identical light pulse conditions (375 nm, 50 mW cm^{-2}, 0.5 s), the response current relaxation time is longer in the device after spin-coating Si QDs, as shown in Fig. S4 (c). This is attributed to the reduced recombination of photogenerated carriers due to their trapping by defect states on the Si QDs surface.

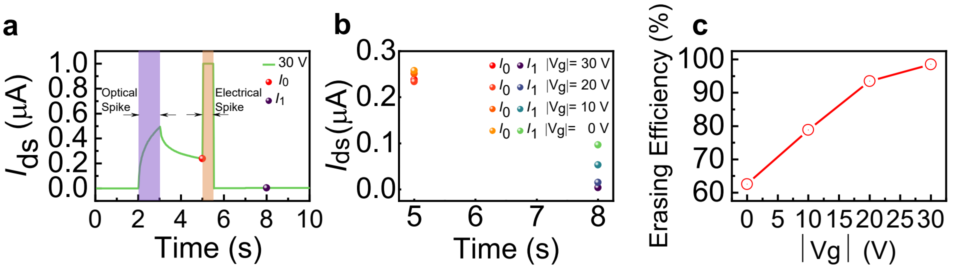

Figure S7. a) Potentiation–depression characteristics of the Si QDs/ReS_2 synaptic device under optical/electrical spike stimuli. b) Initial current (I_0) and post-erase current (I_1) measured under different gate voltages. c) Erase efficiency as a function of the peak amplitude of the gate voltage, defined as $(I_0 - I_1)/I_1$.

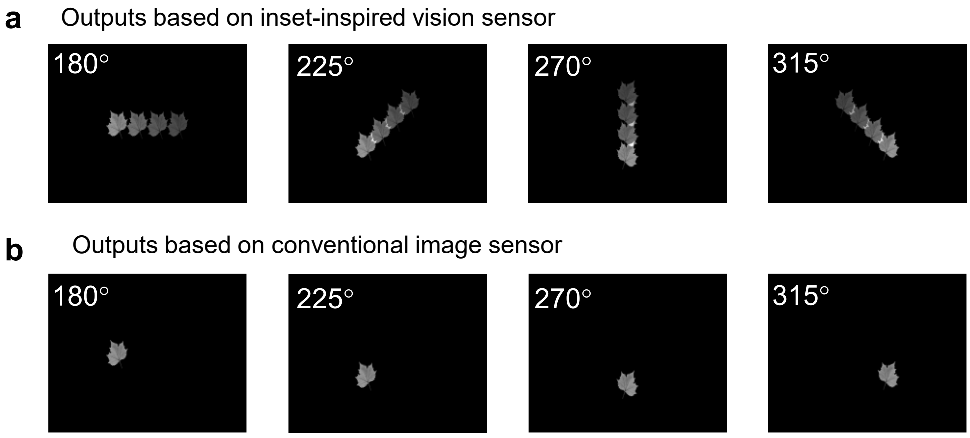

Figure S8. Action recognition based on Si QDs/ReS_2 insect-inspired visual sensors and traditional image sensors. a) and b) display the outputs from the insect-inspired visual sensor and a conventional image sensor, respectively, recorded during leaf motion along four directions (180°, 225°, 270°, 315°).

3D imaging capability demonstration based on full-chip

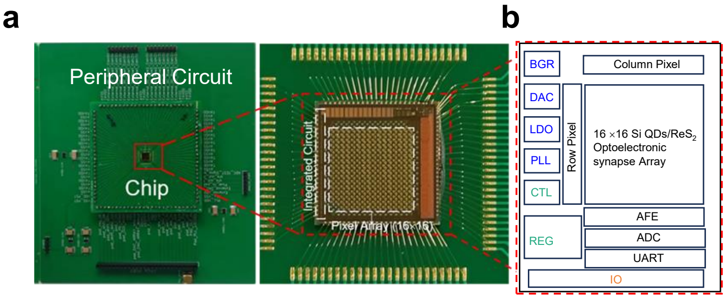

Figure S9. a) Schematic of the Si QDs/ReS_2 heterostructure neuromorphic imaging sensor and its peripheral circuitry. b) Floorplan of the on-chip CMOS integrated circuit showing the relative placement of the constituent modules.

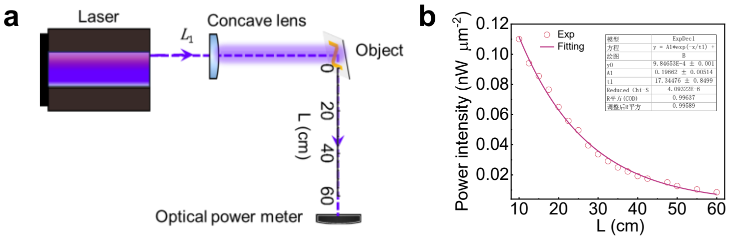

Figure S10. a) Schematic diagram of an experimental setup for investigating the relationship between distance and power intensity. b) The incident light power as a function of distance (L).

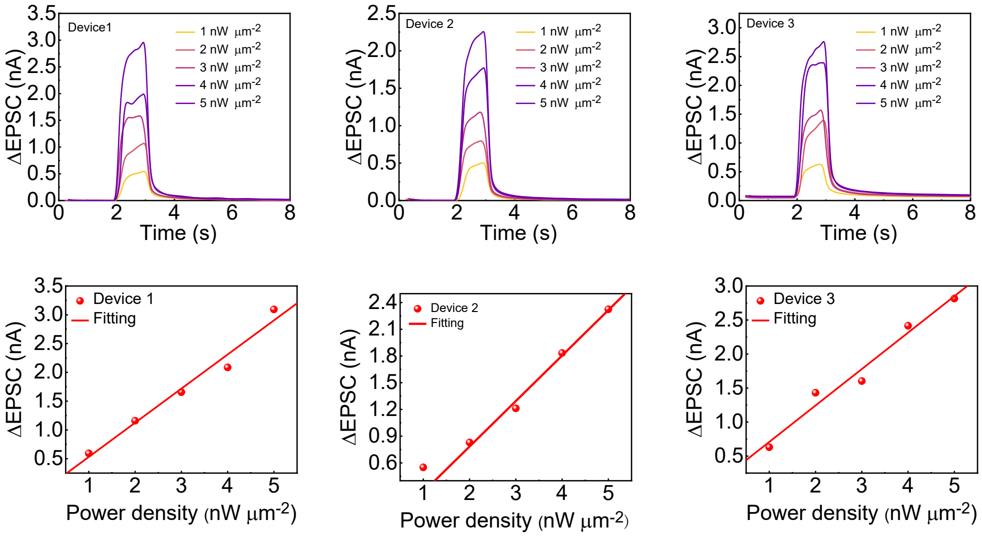

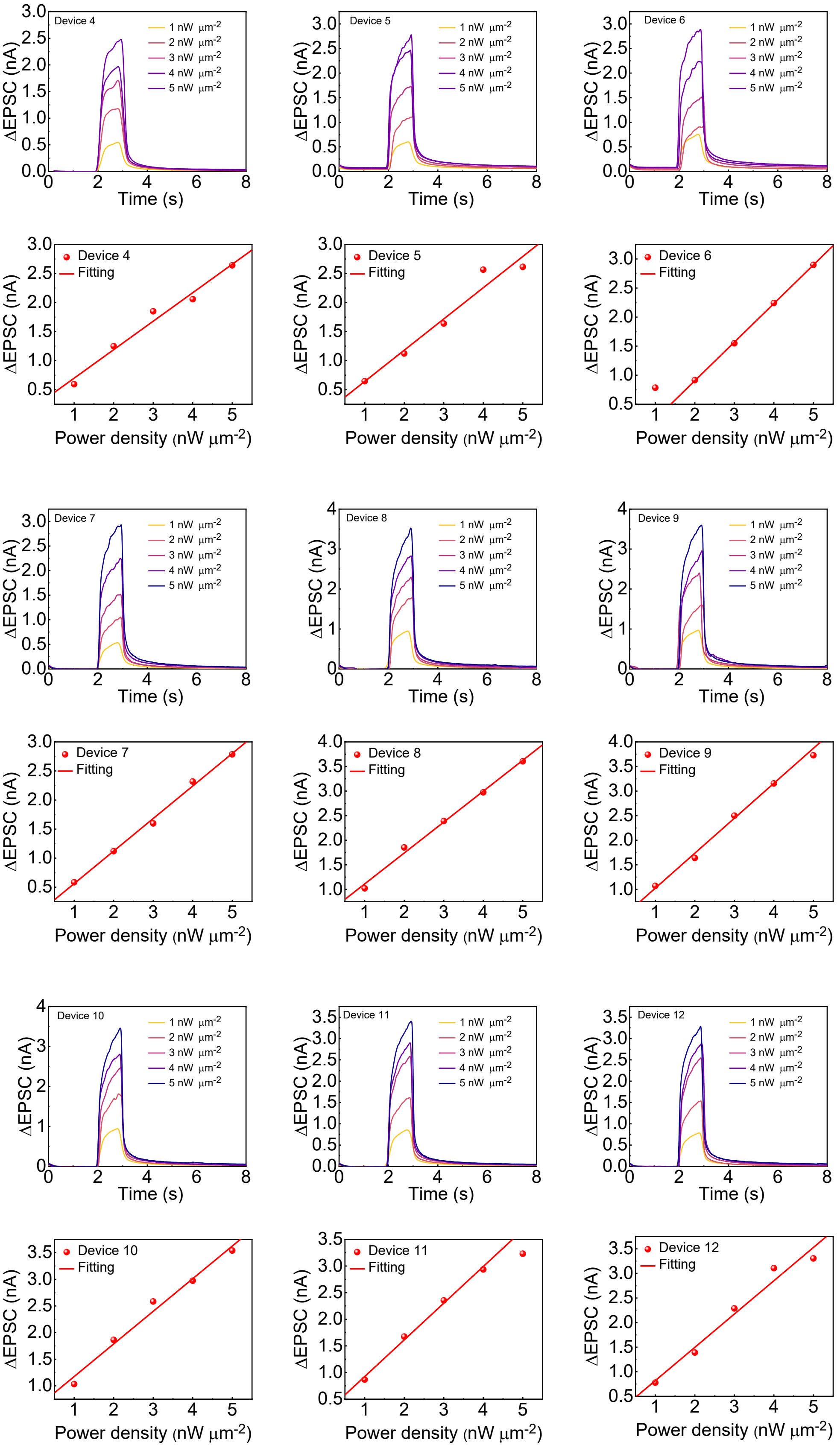
Device 4
Device 5
Device 6
Device 7
Device 8
Device 9
Device 10
Device 11
Device 12
1 nW μm-2
2 nW μm-2
3 nW μm-2
4 nW μm-2
5 nW μm-2
ΔEPSC (nA)
Time (s)
Fitting
Power density (nW μm-2)

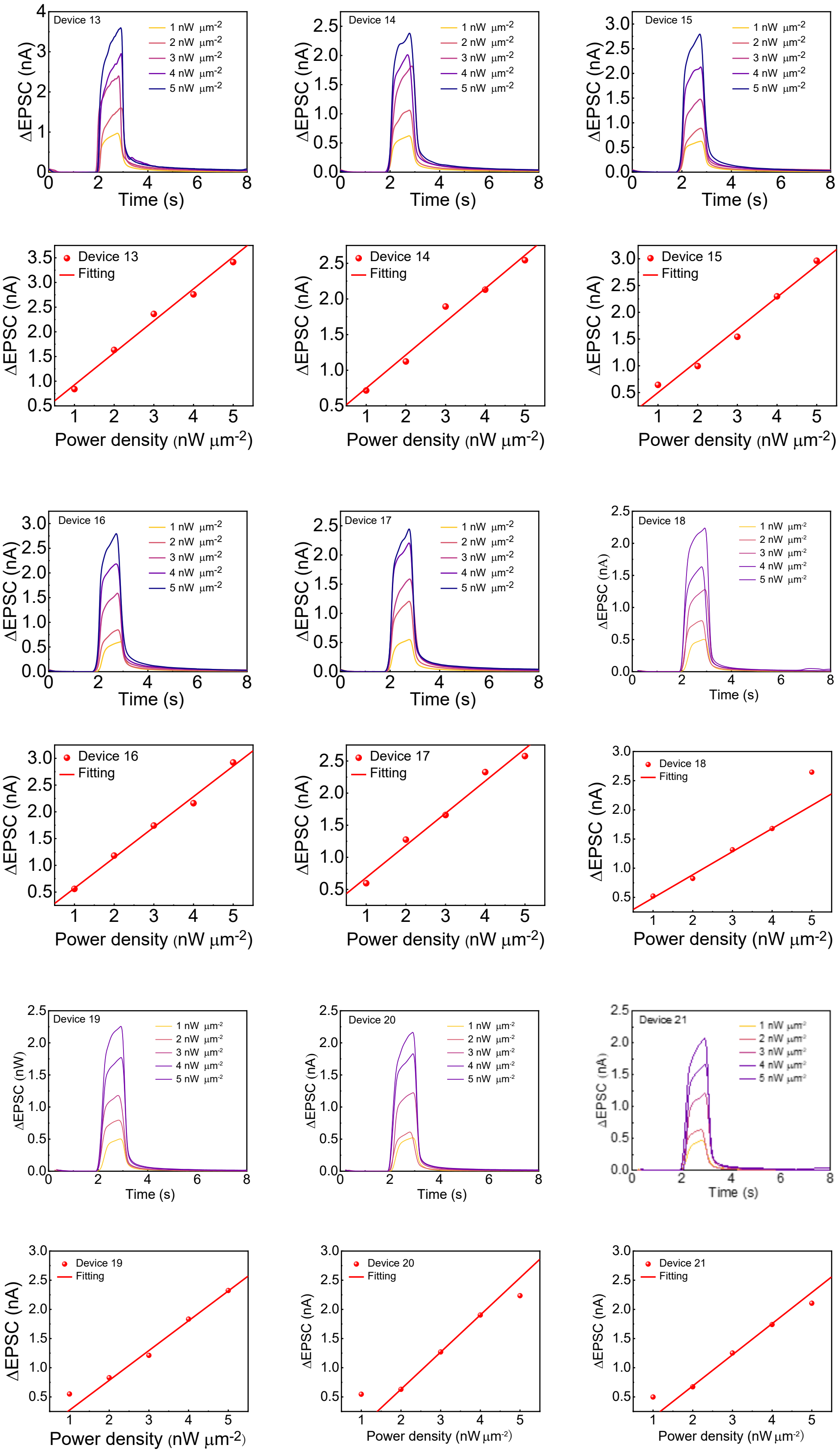
Device 13
Device 14
Device 15
Device 16
Device 17
Device 18
Device 19
Device 20
Device 21
1 nW μm-2
2 nW μm-2
3 nW μm-2
4 nW μm-2
5 nW μm-2
ΔEPSC (nA)
ΔEPSC (nW)
Time (s)
Fitting
Power density (nW μm-2)

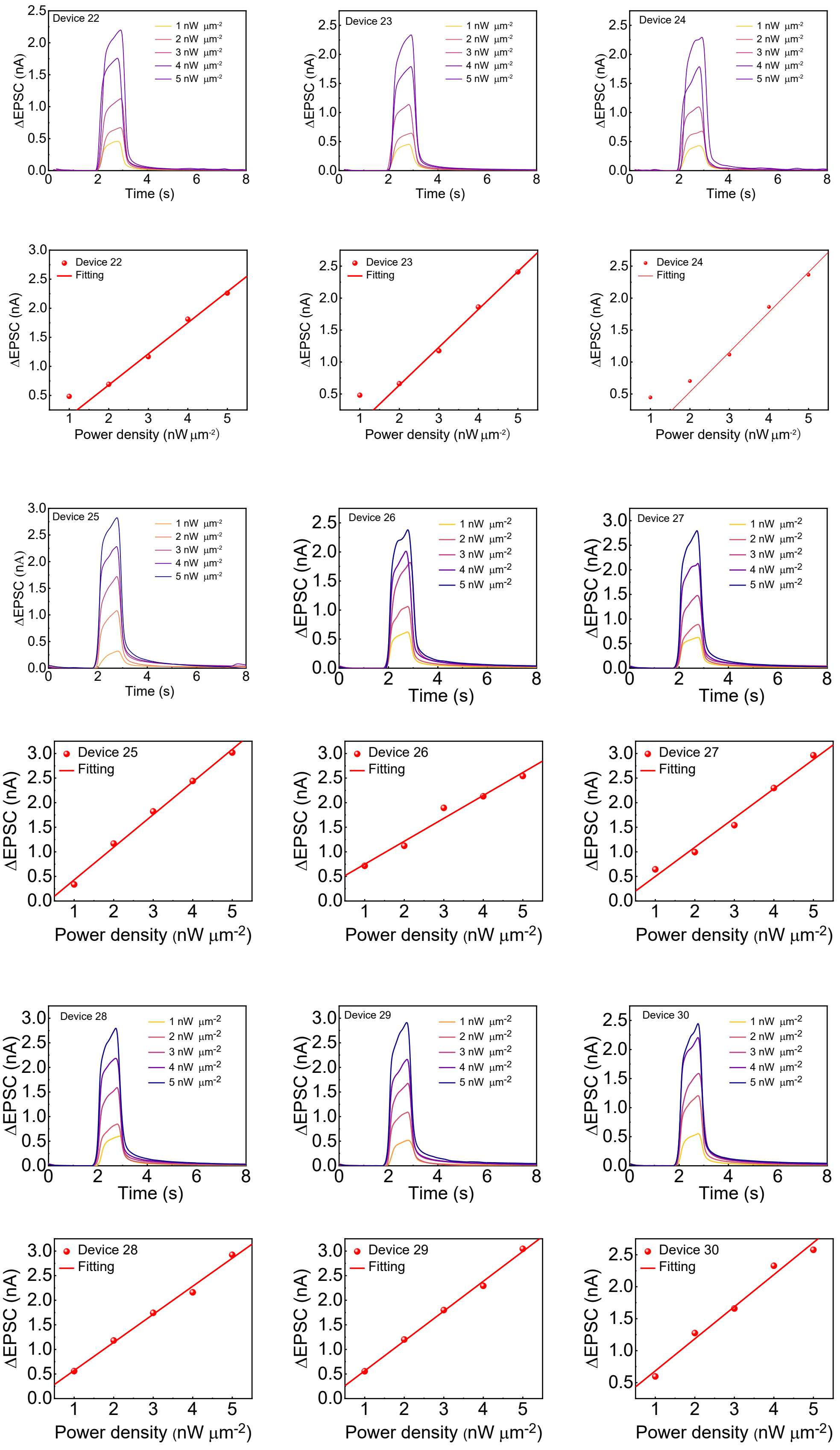

Device 22
Device 23
Device 24
Device 25
Device 26
Device 27
Device 28
Device 29
Device 30
1 nW μm-2
2 nW μm-2
3 nW μm-2
4 nW μm-2
5 nW μm-2
ΔEPSC (nA)
Time (s)
Fitting
Power density (nW μm-2)

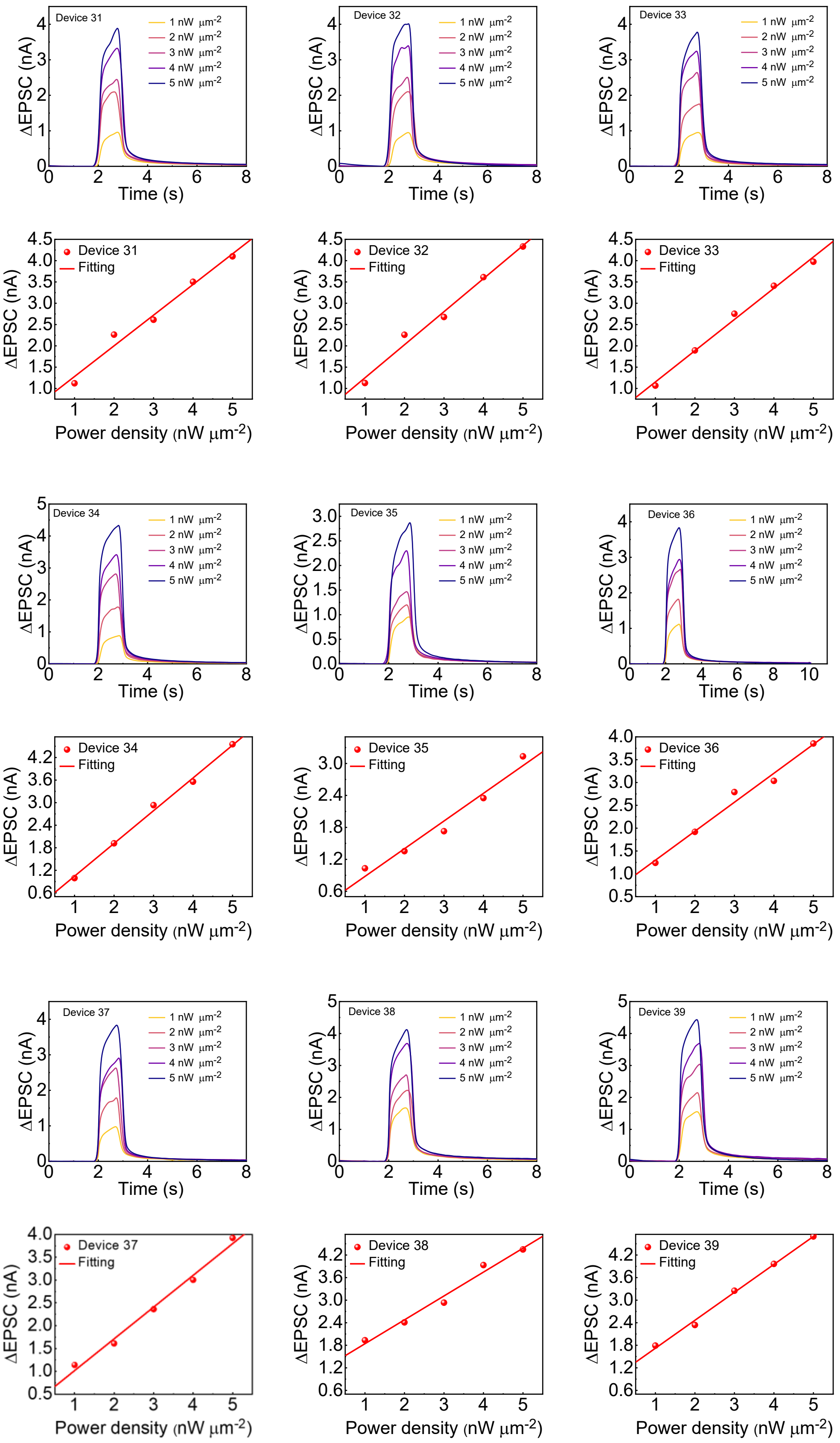
Device 31
Device 32
Device 33
Device 34
Device 35
Device 36
Device 37
Device 38
Device 39
1 nW μm-2
2 nW μm-2
3 nW μm-2
4 nW μm-2
5 nW μm-2
ΔEPSC (nA)
Time (s)
Fitting
Power density (nW μm-2)

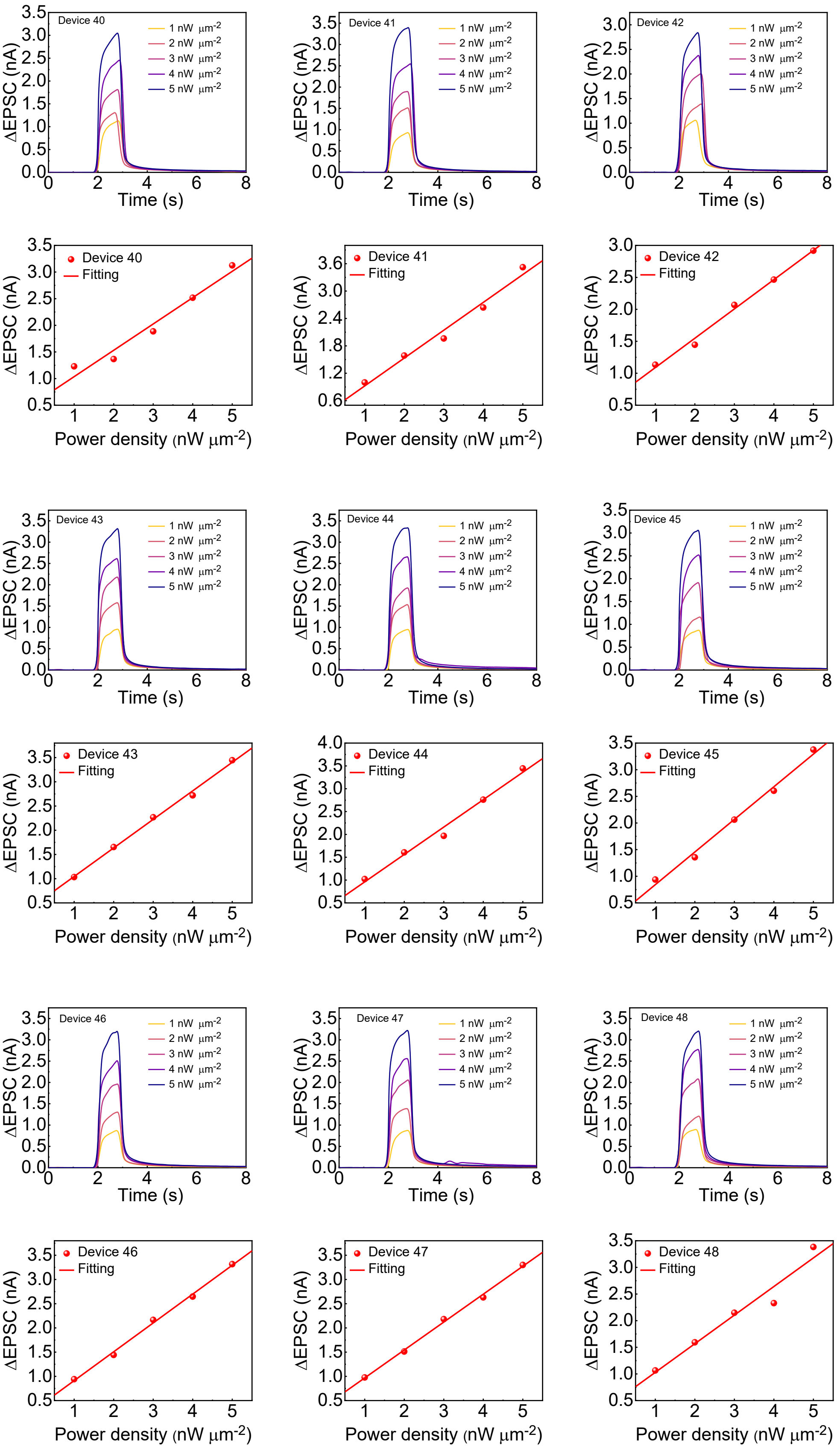
Device 40
Device 41
Device 42
Device 43
Device 44
Device 45
Device 46
Device 47
Device 48
1 nW μm^{-2}
2 nW μm^{-2}
3 nW μm^{-2}
4 nW μm^{-2}
5 nW μm^{-2}
ΔEPSC (nA)
Time (s)
Fitting
Power density (nW μm^{-2})

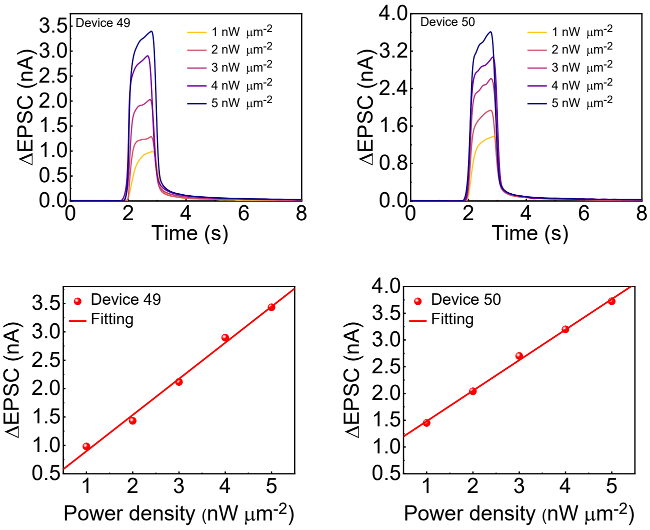

Figure S11. ΔEPSC responses and the fitted relationship between the maximum ΔEPSC and the incident optical power for 50 pixels of a Si QDs/ReS_2 artificial vision chip, measured under 375 nm, 1 s pulsed illumination at varying power levels.

Supplementary Note 1. Calculate the ΔEPSC and normalized conductance of Si QDs/ReS_2 phototransistor synapse

$$\Delta EPSC = I_{\mathrm{ph}} - I_{\mathrm{dark}} \tag{1}$$

$$\text{Normalized conductance} = \frac{G(t_i)}{G(t_0)} \tag{2}$$

Here, I_{dark} denotes the dark current, and ΔEPSC represents the difference between the amplitude of the EPSC following light pulse stimulation and the dark current. G(t_{i}) represents the conductance value of the device at any time. G(t_0) represents the maximum conductance value of the device after excitation by 50 optical pulses at 375 nm.

Supplementary Note 2. Functional characteristics of the CMOS integrated circuit module and its signal flow transmission process

The proposed photodetection chip integrates a monolithically stacked sensing and readout architecture optimized for high-sensitivity optoelectronic imaging. The system comprises four main subsystems: the photodetector array with row/column drivers, an analog front-end (AFE), a 10-bit analog-to-digital converter (ADC), and a data transmitter. Additional support modules include on-chip reference voltage generation, a phase-locked loop (PLL) for system clocking, and a signal generation block for readout control.

At the front-end, the Si QDs/ReS_2 photodetector array serves as the light-sensing unit. Each pixel is individually addressed via integrated row and column drivers and is capacitively coupled to the AFE for low-noise charge amplification. The AFE performs charge integration and amplification while maintaining minimal noise and distortion across the sensing bandwidth.

Following the AFE, a 10-bit successive approximation register (SAR) ADC digitizes the analog signal with sufficient resolution for low-light imaging. The sampling of the AFE and the ADC is precisely timed by a dedicated signal generation module, which orchestrates the readout process to maximize dynamic range and throughput.

A serializer and data transmitter module streams out the digitized image data off-chip. Meanwhile, on-chip voltage reference generators and a PLL module ensure low-jitter clock generation and stable biasing conditions for all analog and digital submodules.

The tight integration of the optoelectronic front-end with mixed-signal processing and timing control circuits enables compact, low-power, and high-performance imaging on a single chip.

Supplementary Note 3. Relationship between distance and power intensity

To test the variation of light intensity with distance, we take an opaque object "Z" and place a 375nm laser parallel to the front of the object, keeping the excitation power constant. Then the laser emitted by the laser is reflected by the mark, and the light intensity of the reflected light is measured using an optical power meter. By changing the distance between the optical power meter and the mark, the light intensity of the reflected light at the corresponding distance is measured, and the relationship between the light intensity and the distance obtained is shown in Figure S4a.

We find that the power intensity (P_{in}) of scattered light on an object decreases with distance L, and the relationship between L and P_{in} is obtained by fitting: $P_{\mathrm{in}} \propto ae^{b\times L}$. Therefore, at a constant laser intensity of 375 nm, the Si QDs/ReS_2 artificial vision chip receives an optical signal intensity P_{in} that varies with L. We recorded the EPSC response of the corresponding pixel point of the Si QDs/ReS_2 artificial vision chip after emitting a light pulse with a duration of 1 s to the object, and varied the distance between the Si QDs/ReS_2 artificial vision chip and the object to obtain other data, and We recorded the intensity (P_{in}) of the light corresponding to each distance X(t). Figure S5 shows the light impulse response of the corresponding 50 pixels on the Si QDs/ReS_2 artificial vision chip for the ΔEPSC with P_{in}. The relationship between the max value of ΔEPSC and P_{in} as the equation: $\Delta EPSC = c \times P_{in} + d$ (c and d are constants, respectively), so the relationship between the max value ΔEPSC and distance X(t) can be correlated with L as $\Delta EPSC \propto \alpha ce^{b\times X(t)} + d$.
